# Dose escalation study of targeted alpha therapy with [^225^Ac]Ac-DOTA-substance P in recurrence glioblastoma – safety and efficacy

**DOI:** 10.1007/s00259-021-05350-y

**Published:** 2021-04-15

**Authors:** Leszek Królicki, Frank Bruchertseifer, Jolanta Kunikowska, Henryk Koziara, Dariusz Pawlak, Radosław Kuliński, Rafał Rola, Adrian Merlo, Alfred Morgenstern

**Affiliations:** 1 Department of Nuclear Medicine, Medical University of Warsaw, ul. Banacha 1 a, 02-097 Warsaw, Poland; 2 European Commission, Joint Research Centre, Directorate for Nuclear Safety and Security, Karlsruhe, Germany; 3 Department of Neurosurgery, Maria Sklodowska-Curie National Research Institute of Oncology, Warsaw, Poland; 4 Radioisotope Centre POLATOM, National Centre for Nuclear Research, Otwock, Poland; 5 Department of Neurology, Military Institute of Aviation Medicine, Warsaw, Poland; 6 Neurosurgical Center Berne and University of Basel, Basel, Switzerland

**Keywords:** Glioblastoma, Alpha therapy, Substance P, Actinium, ^225^Ac, ^68^Ga, TAT, SP, GBM

## Abstract

**Abstract:**

Glioblastoma is the most common and malignant primary brain tumour, with a poor prognosis. Introduction of new treatment options is critically important. The study aimed to assess the appropriateness of escalation doses and toxicity of [^225^Ac]Ac-DOTA-SP therapy.

**Material and methods:**

A total of 21 patients (age of 43.0 ± 9.5 years), with histologically confirmed recurrent or conversion glioblastoma grade 4 following a standard therapy, have been included in the study. One to 2 intracavitary port-a-cath systems were stereotactically inserted. Patients were treated with escalation dose protocol with 10, 20 and 30 MBq per cycle totally 1–6 doses of [^225^Ac]Ac-DOTA-SP in 2-month intervals. Therapeutic response was monitored by clinical performance status and MRI imaging.

**Results:**

Treatment was well tolerated with mostly mild temporary adverse effects (oedema, epileptic seizures, aphasia, hemiparesis) mainly in the group of patients treated with 30 MBq of [^225^Ac]Ac-DOTA-SP. Only one patient treated with 30 MBq revealed thrombopenia grade 3. There was no other grade 3 and 4 toxicity related to [^225^Ac]Ac-DOTA-treatment in all groups. The median overall survival time from the primary diagnosis (OS-d) was 35.0 months and from the diagnosis of the recurrence/conversion (OS-r/c) was 13.2 months. From the start of treatment with [^225^Ac]Ac-DOTA-SP, the median PFS was 2.4 months, and the OS-t was 9.0 months. There were no statistically significant differences between the investigated dose escalation groups.

**Conclusions:**

Treatment of recurrent glioblastoma with [^225^Ac]Ac-DOTA-SP is safe and well tolerated up to 30 MBq per cycle. The escalation dose protocol showed good tolerability. Only mild temporary adverse effects were observed. No remarkable haematological, kidney and liver toxicity was seen.

## Introduction

Glioblastoma multiforme (GBM), lately according to the World Health Organization Classification of Tumours of the Central Nervous System from 2016, called glioblastoma [1], is the most common and malignant primary brain tumour. While it is more prevalent among older adults, this disease could appear in people of all ages. Histological classification is strongly associated with patient age. Typically, older patients are more likely to have primary glioblastoma, and they present more aggressive clinical behaviour with rapid growth. Secondary glioblastomas are more often associated with younger patients who frequently have an established history of lower-grade gliomas (grade 2–3).

In both scenarios, the current standard of care involves maximal safe surgical resection followed by concurrent chemotherapy with radiation followed by adjuvant chemotherapy. Unfortunately, diagnosed patients in the majority have a recurrence of disease and die within a 1.5–2-year treatment schedule. The therapeutic limitations in these malignancies are caused by the infiltrative nature of the glioma cells. The prognosis of patients with recurrent glioblastoma is even more serious, and the median overall survival in this group is less than 6 months [2, 3].

Therefore, introduction of new treatment options is critically important for this group of patients. Regardless of the histological grading, glioma cells are characterized by a high expression of NK-1 receptors which bind the natural ligand substance P (SP). SP, initially labelled with beta emitters and then followed with alpha emitters, have been used for locoregional application into the tumour. Local application allows to directly deliver highly active radiopharmaceuticals into a malignant brain tumour, circumventing the blood-brain barrier and reducing systemic adverse effects.

Initially, glioblastoma patients were treated locally with the beta emitter [^90^Y]Y-DOTA-SP [4]. In the majority of patients, the authors observed disease stabilization and/or improved neurologic status with a median OS after the initiation of local treatment of 11 months and a median OS of 16 months. Only transient toxicity was seen as symptomatic radiogenic oedema in one patient.

Based on the promising outcome of these initial clinical experiences with a locoregional injection of beta emitters, alpha emitters were also included into the clinical protocol. Alpha particles have many advantages over beta particles. First of all, the energy is higher with short tissue range penetration of less than 100 μm; secondly, due to the direct radiation effect, hypoxia and cell cycle are not critical to the effect of radiation. Initially, ^213^ Bi was used for labelling of SP; bismuth-213 is convenient for labelling of peptides, and the short *T*_1/2_ of ^213^Bi (*T*_1/2_ = 46 min) is beneficial for radiation protection.

A pilot study including 5 patients treated with ^213^Bi-[Thi^8^, Met(O_2_)^11^]-substance P ([^213^Bi]Bi-DOTA-SP) showed good tolerability, and MRI revealed radiation-induced necrosis and demarcation of the tumours [5]. Therefore, the study concluded that targeted local radiotherapy with [^213^Bi]Bi-DOTA-SP may be an effective treatment for critically located malignant gliomas.

Based on this initial experience, treatment with [^213^Bi]Bi-DOTA-SP was continued in collaboration with JRC Karlsruhe and Medical University Warsaw [6, 7].

The first published study described the therapeutic outcome in the recurrence of secondary glioblastoma [6]. In this group, the median time from the first diagnosis to the tumour transformation into glioblastoma was 18.4 months. The median OS time from the first diagnosis (OS-d) was 52.3 months; the median OS from conversion (OS-c) was 18.6 months. From the onset of [^213^Bi]Bi-DOTA-SP treatment, the median PFS was 5.8 months, and the median OS (OS-t) was 16.4 months.

The second publication included 20 patients with histologically confirmed recurrent glioblastoma grade IV after standard therapy [7]. The median overall survival from the first diagnosis was 23.6 months, and the median survival after recurrence was 10.9 months. The median survival time from the start of [^213^Bi]Bi-DOTA-SP was 7.5 months.

However, although the results look promising, the short half-life of ^213^Bi (*T*_1/2_ = 46 min) might compromise the delivery of sufficient doses to remote tumour cells. Therefore, ^225^Ac has recently been introduced due to its increased cytotoxicity as well as a longer *T*_1/2_ (9.9 days) which should enlarge the delivery dose of the radiopharmaceutical within the tumour.

This paper presents the first experience of targeted alpha therapy with radiolabelled [^225^Ac]Ac-DOTA-SP in 21 patients with recurrent primary and secondary glioblastoma grade IV. The aim of this study was to develop a treatment protocol for [^225^Ac]Ac-DOTA-SP alpha-therapy. The primary end point was to assess the appropriateness of escalation doses and toxicity of the approach. The secondary end point was to report the outcome of therapy in terms of the progression-free survival (PFS) and overall survival (OS).

## Materials and methods

[^225^Ac]Ac-DOTA-SP treatment was performed as salvage therapy in patients with recurrence glioblastoma WHO grade IV, primary and secondary. The examined group consists of 21 patients (13 males and 8 females) with an average age of 43.0 ± 9.5 years, following a standard therapy (surgery, radio- and chemotherapy) performed in the period 2013–2020. Patients were informed about the experimental nature of the therapy and gave written informed consent. The study was approved by the Ethical Committee of the Medical University of Warsaw (KB/235/2011 and KB/172/2016).

The following inclusion criteria were applied:

- Histopathologically confirmed recurrent glioblastoma tumour, grade 4 (WHO)
- Tumour volume below 90 mL as defined by the T1-weighed contrast-enhanced MRI
- Absence of obstruction of CSF circulation or decompensating intracranial pressure
- Karnofsky performance score > 40
- No pregnancy or lactation
- Age higher than 18 years, absence of psychological, familial and sociological conditions potentially hampering compliance with the study protocol

### MRI protocol

MRI examinations were performed using GE Excite HD 1.5 T (GE Healthcare, USA) or Siemens 3 T MRI scanner (Siemens Medical Solutions Inc., USA).

MRI protocol before intravenous gadolinium-based contrast agent administration included three-dimensional T1-weighted images (3D-T1w), axial bi-dimensional T2-Fluid-attenuated inversion recovery (FLAIR) images, and axial bi-dimensional diffusion-weighted imaging (DWI) and, after contrast administration, axial bi-dimensional T2-weighted and three-dimensional T1-weighted images. Imaging parameters are DWI:TR/TE, 4900/92 ms; FOV, 220 mm × 220 mm; slice thickness, 5 mm; matrix, 160 × 160; and b values of 500–1000 mm^2^/s. The apparent diffusion coefficient (ADC) was calculated automatically by the software and then displayed as a parametric map. ADC measurements were recorded for a given region by drawing regions of interest (ROIs) on the ADC map.

The images were viewed and interpreted by two experienced radiologists.

### Radionuclides and radiolabelling

The DOTA-[Thi^8^, Met(O_2_)^11^]-substance P was obtained from Bachem, Switzerland, and labelled with ^68^Ga (^68^Ge/^68^Ga generators GalliaPharm generator, Eckert & Ziegler, Germany) as described previously [6, 7].

^225^Ac was obtained by radiochemical extraction from ^229^Th sources at the Directorate for Nuclear Safety and Security of the Joint Research Centre of the European Commission in Karlsruhe, Germany [8].

The labelling of therapeutic doses of [^225^Ac]Ac-DOTA-SP was performed in 0.1 M TRIS buffer using a microwave synthesizer (Biotage® Initiator) at 95 °C for 5 min. Quality control was performed by instant thin-layer chromatography (Tec Control 150-771; Biodex Medical Systems) and 0.5-M sodium citrate as a developing agent.

Radiochemical purity of the radiopharmaceutical assessed by ITLC was more than 98.0% and specific activity for [^225^Ac]Ac-DOTA-SP up to 1 MBq/nmol. The pH was adjusted to 6.5–7.5 with ascorbic acid.

Sterility of the final formulation was ensured via sterile filtration (Millex-GV, 0.20 μm; Millipore). Before injection, 10 MBq of [^68^Ga]Ga-DOTA-SP was added to [^225^Ac]Ac-DOTA-SP, allowing for control of local injection by PET/CT imaging.

### Study protocol and injection of the radiopharmaceutical

One or two catheters connected to a subcutaneous port (Medtronic, USA) were implemented stereotactically into the postsurgical cavity 2–4 weeks before treatment. The proper catheter position and lack of ventricular connection were confirmed in MRI in procedure previously described [6, 7].

Because safety and tolerability of local usage of the radiopharmaceutical [^225^Ac]Ac-DOTA-SP in glioma patients had not been described previously, a dose escalation scheme has been assessed starting at an activity of 10 MBq followed by 20 MBq and 30 MBq.

Patients were treated with 1–6 cycles of [^225^Ac]Ac-DOTA-SP in 2-month intervals, depending on the clinical condition.

The injection procedure of [^225^Ac]Ac-DOTA-SP and PET/CT after co-injection of [^68^Ga]Ga-DOTA-SP with therapeutic doses of [^225^Ac]Ac-DOTA-SP were performed as a previous described protocol for [^213^Bi]Bi-DOTA-SP [6, 7].

Briefly, the port was punctured with a butterfly needle. Following a control injection of 1 mL of saline, the active drug was injected in the volume of 2 mL. The injection velocity was 0.5 mL/min.

### Toxicity and follow-up

The monitoring of the [^225^Ac] activity in the blood and urine was performed in 5 patients, at different time points 30 min up to 2 days after the injection depending on the availability. For the assessment of the [^225^Ac] activity, aliquots of 1 ml were collected, and the [^221^Fr] daughter gamma line at 218 keV or the [^213^Bi] gamma line was used after reaching the appropriate equilibrium (minimum 45 min after sampling for [^221^Fr] and minimum 4 h after sampling for [^213^Bi]) by using either a high-resolution gamma detector system or NaI gamma detection system. The calculation of the dose was half-life corrected to the time of injection.

Laboratory test including blood cell count, haemoglobin, creatinine, blood-urea-nitrogen, liver enzymes, clinical status, and patient history was done every 4–6 weeks after each cycle of treatment and classified into toxicity grades using the Common Terminology Criteria for Adverse Events v3.0 (CTCAE). Imaging MRI follow-up was performed every 4–6 weeks post each injection and thereafter every 3 months and compared to baseline.

### Statistical methods

Parameters were characterized by the mean (± SD). Progression-free survival time (PFS) was defined as the time from the start of radioisotope treatment to the first evidence of progression or relapse, or to death. The 6-month OS (OS6) and 12-month OS (OS12) rate was defined as the number of subjects who did not die prior to 6 months/12 months from the date of their first dose of [^225^Ac]Ac-DOTA-SP, divided by the number of subjects in the cohort.

The overall survival from the diagnosis was defined as the time from the first diagnosis of the tumour to death from any cause (OS-d); OS from recurrence or conversion was defined as the time from the diagnosis of recurrence in primary tumours or conversion in secondary, to death from any cause (OS-r/c). OS from the start of treatment was defined as the time from the first cycle of [^225^Ac]Ac-DOTA-SP treatment to death from any cause (OS-t). Survival parameters were calculated using the Kaplan-Meier estimator and compared using the log-rank test. Calculations were performed using GraphPad PRISM 5 (GraphPad Software Inc).

## Results

### Patient characteristics and functional status

MRI of the brain demonstrated recurrence of disease in the form of diffuse T2 and FLAIR changes and irregular ring-shaped contrast enhancement part in T1 around the postoperative cavity. The median viable tumour volume measured in MRI (defined on contrast-enhanced T1 image) was 35.7 mL (ranged from 4.5 to 83 mL). The median volume of post-operative cavity was 14.8 (ranged from 0.5 to 82.1 mL).

The functional status of patients before the treatment and during the follow-up was assessed using the Karnofsky status and Barthel Index. The median pre-therapeutic Karnofsky status was 70 (ranged from 40 to 100) and Barthel Index 90 (ranged from 25 to 100).

With 10 MBq of [^225^Ac]Ac-DOTA-SP, four patients were treated with 1–5 cycles (1 cycle in 1 patient, 2 cycles in 1 patient, 4 cycles in 1 patient, 5 cycles in 1 patient).

With 20 MBq of [^225^Ac]Ac-DOTA-SP ten patients were treated with 1–6 cycles (1 cycle in 4 patients, 3 cycles in 2 patients, 4 cycles in 1 patient, 5 cycles in 2 patients, 6 cycles in 1 patient).

With 30 MBq of [^225^Ac]Ac-DOTA-SP, seven patients were treated with 1–3 cycles (1 cycle in 2 patients, 2 cycles in 4 patients, 3 cycles in 1 patient). Detailed patient characteristics are summarized in Table 1 and divided by group in Table 2.

**Table 1:** Detailed patient characteristics

	Age at start of Ac therapy	Grade 4	Total (MBq)	Single injection group	Cycles	Barthel	Karnofsky	Before treatment viable tumour vol	ADC (mm^2^/s)
1	41	Primary glioblastoma NOS	53.4	10	5	100	80	27.3	nd
2	42	Primary glioblastoma NOS	44.8	10	4	100	90	23.1	341
3	64	Primary glioblastoma NOS	22.8	10	2	100	80	64.3	nd
4	48	Primary glioblastoma NOS	11.6	10	1	60	60	20.4	213
5	43	Secondary glioblastoma NOS	101.5	20	5	100	100	4.5	1007
6	43	Primary glioblastoma IDH wildtype	149.5	20	6	90	70	53.2	1398
7	39	Secondary glioblastoma IDH wildtype	98.7	20	5	45	40	72.8	nd
8	38	Secondary glioblastoma NOS	97.0	20	4	90	70	29.2	nd
9	32	Primary glioblastoma NOS	17.6	20	1	90	60	83.0	nd
10	48	Primary glioblastoma NOS	57.6	20	3	70	70	42.7	nd
11	63	Primary glioblastoma NOS	18.1	20	1	25	50	42.7	314
12	53	Primary glioblastoma NOS	19.1	20	1	80	100	26.6	nd
13	28	Primary glioblastoma NOS	20.6	20	1	85	70	57.6	360
14	45	Primary glioblastoma NOS	75.0	20	3	100	90	42.1	856
15	42	Primary glioblastoma IDH mutant	105.0	30	3	95	80	23.0	1770
16	37	Secondary glioblastoma IDH mutant	54.6	30	2	100	80	62.4	985
17	44	Secondary glioblastoma IDH mutant	26.8	30	1	65	60	10.3	825
18	35	Primary glioblastoma NOS	53.2	30	2	100	80	24.8	1362
19	46	Primary glioblastoma NOS	54.9	30	2	80	60	17.7	1095
20	26	Secondary glioblastoma NOS	56.5	30	2	75	60	6.5	1103
21	47	Primary glioblastoma NOS	28.4	30	1	100	90	77.3	1423

*nd, no data

*ADC*, apparent diffusion coefficient

**Table 2:** Patient’s data in groups treated with 10 MBq, 20 MBq and 30 MBq of [^225^Ac]Ac-DOTA-SP

	10 MBq (*n* = 4)	20 MBq (*n* = 10)	30 MBq (*n* = 7)
Mean age (years)	48.8 ± 10.6	43.2 ± 10.1	39.6 ± 7.5
Mean tumour volume (ml)	33.8 ± 20.6	45.4 ± 22.8	31.7 ± 27.2
Mean number of cycles	3 ± 1.8	3 ± 1.9	1.9 ± 0.7
Mean total injected activity (MBq)	32.2 ± 19.3	65.5 ± 46.3	54.2 ± 25.8

### [^68^Ga]Ga-DOTA-SP post-therapeutic imaging

To study the whole-body distribution of [^225^Ac]Ac-DOTA-SP, post-therapeutic scans were performed. All cases, [^68^Ga]Ga-DOTA-SP PET/CT presented increased uptake in the tumour area and very low distribution in the kidney and urine. This image was also used for quality control of injection. Out of a total of 55 injections, 5 were in part inaccurate because of 3 partially subcutaneous injections, one with partial connection to the ventricles and one catheter occlusion.

**Fig. 1:**
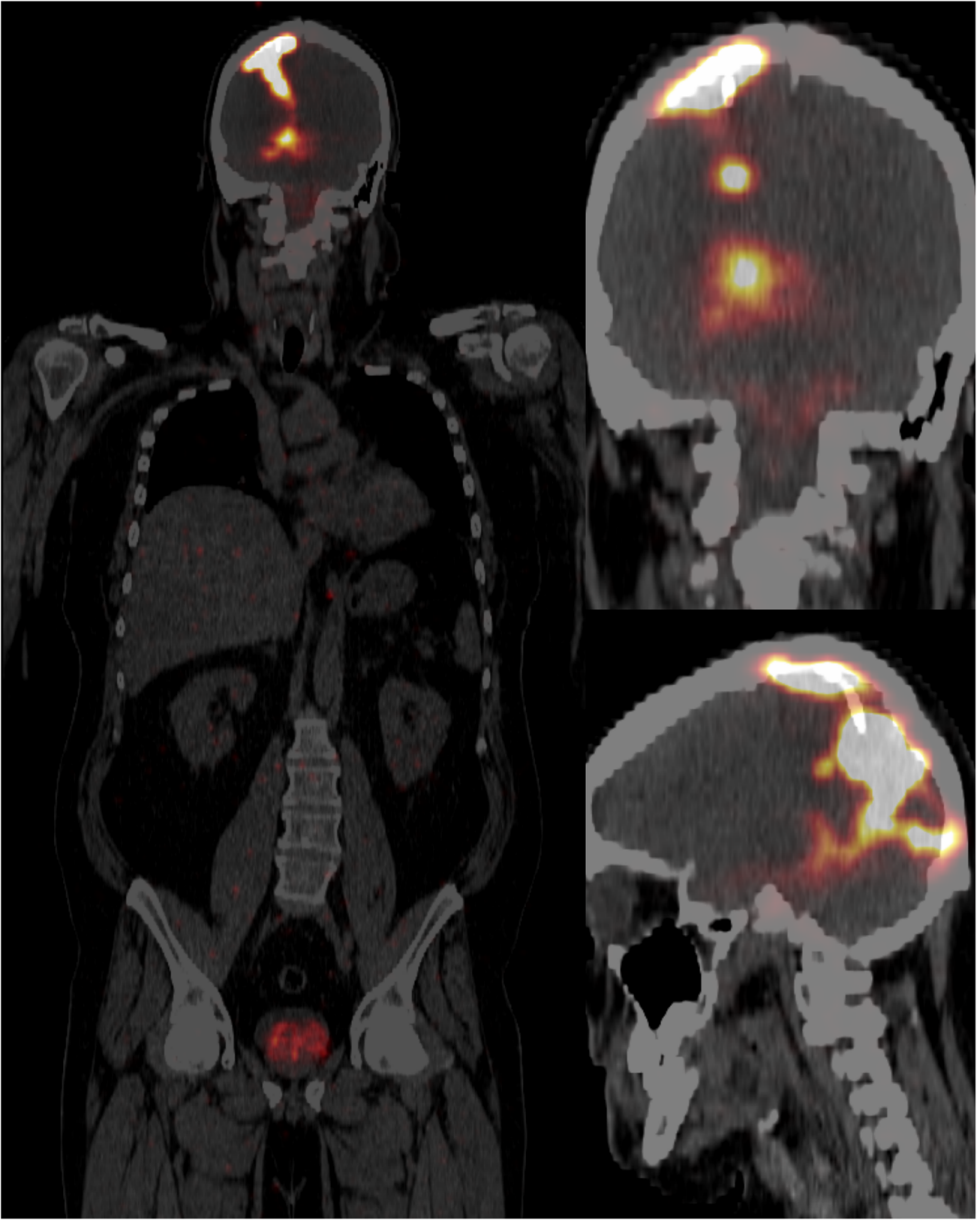
PET/CT after local co-injection of 10-MBq [^68^Ga]Ga-DOTA-SP with a therapeutic dose of [^225^Ac]Ac-DOTA-SP into the resection cavity of a glioblastoma with leakage to the ventriculus. The activity in the bladder was 2.5%

**Fig. 2:**
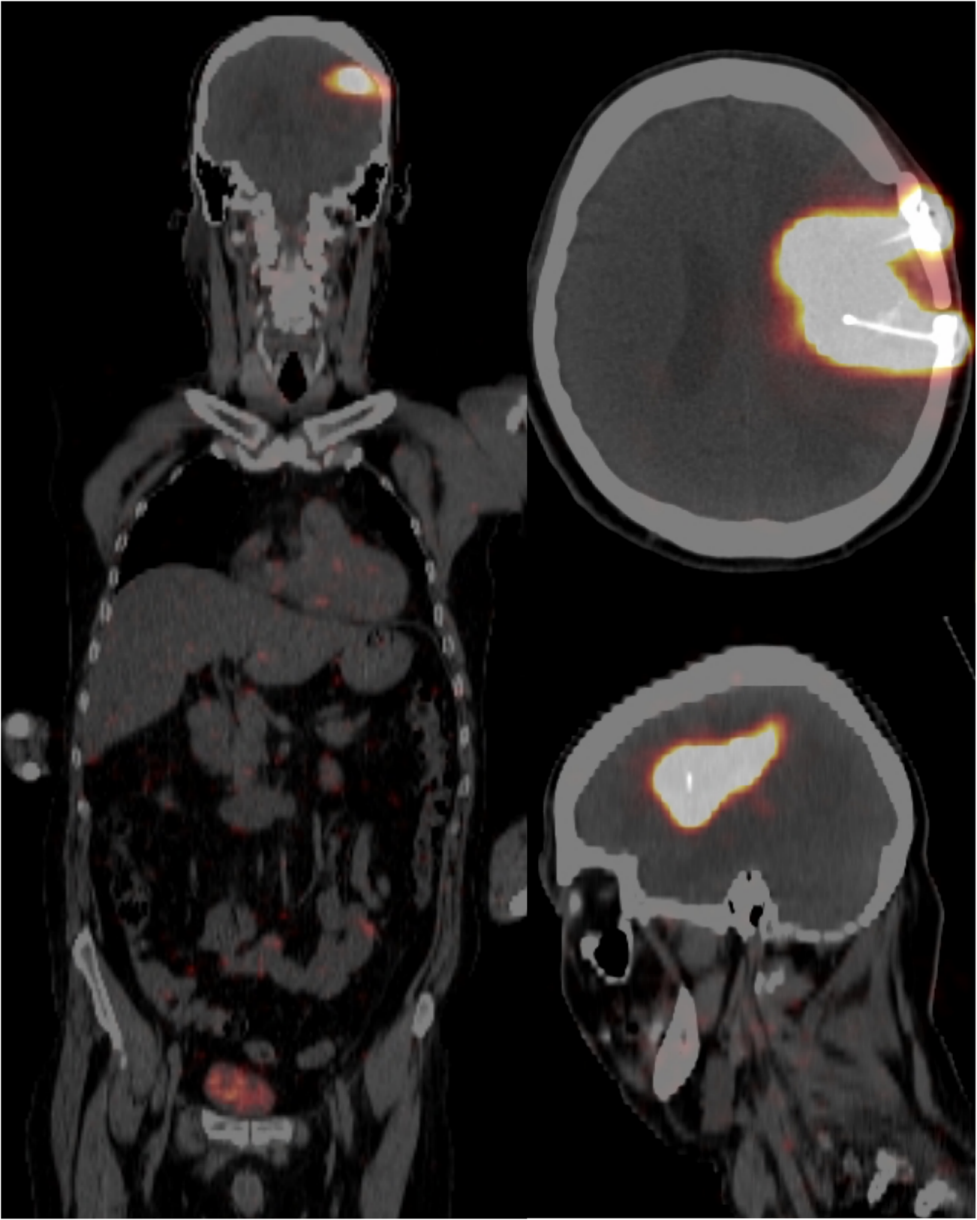
PET/CT after local co-injection of 10-MBq [^68^Ga]Ga-DOTA-SP with a therapeutic dose of [^225^Ac]Ac-DOTA-SP into the resection cavity of a glioblastoma. Most of the activity is concentrated within the lesion; little activity is remaining in the capsule and within the catheter. The activity in the bladder was <1%

In the event of incorrect injection, the activity measured in the bladder was more than 1% with a range of 1.1–3.6%. In all other cases, this activity was less than 1% with a median of 0.3% (range: 0.1–0.9). Examples of biodistribution in the case of correct and incorrect injection are shown in Figs. 1 and 2.

Consequently, activities found in the blood pool typically corresponded to less than 3% of the activities injected intratumorally and at 1 h post injection.

### Toxicity and side effects of treatment with [^225^Ac]Ac-DOTA-SP

Generally, local treatment with [^225^Ac]Ac-DOTA-SP was well tolerated in all investigated dose groups. In all patients, haematotoxicity, nephrotoxicity and hepatotoxicity factors were analysed.

In the first group treated with 10 MBq of [^225^Ac]Ac-DOTA-SP per cycle, no toxicity was seen at all.

In the second and third group treated with 20 MBq and 30 MBq of [^225^Ac]Ac-DOTA-SP, we observed haematological toxicity: thrombopenia grade 2 in one patient in the 20-MBq group and thrombopenia grade 3 in one patient in the 30-MBq group.

The patient with thrombopenia grade 2 had previously documented severe haematological toxicity during chemotherapy which led to the withdrawal of this treatment. [^225^Ac]Ac-DOTA was started 2 months later. In the patient with thrombopenia grade 3, chemotherapy using temozolomide and vincristine had been added to [^225^Ac]Ac-DOTA treatment because of the manifestation of a new focal satellite lesion distant from the targeted area revealed by follow-up MRI during treatment.

Hepatic toxicity grade 1 was seen in one patient of the 30-MBq dose escalation group. Liver dysfunction treated with hepatoprotective drugs had previously been observed following chemotherapy. In the follow-up, symptoms of metabolic syndrome including elevated fasting blood glucose were presented. Liver parameters have stabilized after reducing the dose of steroids. Toxicity appears to have been due to drugs taken during isotopic therapy.

There was no other toxicity observed related to [^225^Ac]Ac-DOTA-SP in the 3 dose groups.

According to neurological status, epilepsy before radioisotope treatment was noted in 9 patients.

No neurological symptoms had been observed in the 10-MBq [^225^Ac]Ac-DOTA-SP group.

In the group treated with 20 MBq [^225^Ac]Ac-DOTA-SP, five patients had epilepsy before radioisotope treatment, and in one of them, 5 days after injection, epileptic seizures were observed. Seven patients had focal neurological symptoms (hemiparesis, 4; aphasia, 3); three of them revealed only transient worsening of hemiparesis or aphasia, but in one of them, local symptoms were progressive. In another patient, transient worsening of the general clinical condition was observed.

In the group treated with 30-MBq [^225^Ac]Ac-DOTA-SP, four patients presented epileptic seizures before radioisotope treatment, and in three of them, 2–5 days after injection, epileptic attacks were noted. Four patients from this group had hemiparesis before radioisotope treatment, and in three of them, transient worsening of symptoms was observed.

In two patients 2 and 3 weeks after treatment with 30 MBq of [^225^Ac]Ac-DOTA-SP, severe perifocal brain oedema was observed, required hospitalization and intensive iv treatment, without intubation.

In the entire study group, 8 patients had satellite glial tumours diagnosed 2–3 months after the start of treatment. In three patients from this group – as a salvage treatment – second cath-path system was implanted, and radioisotope treatment was continued in both places.

### Therapeutic outcome

At the time of analysis, six patients are still alive, and 15 patients have died due to disease progression.

The time from the primary diagnosis OS-d was 35.0 months, and the time from the diagnosis of the recurrence OS-r/c was 13.2 months.

**Fig. 3:**
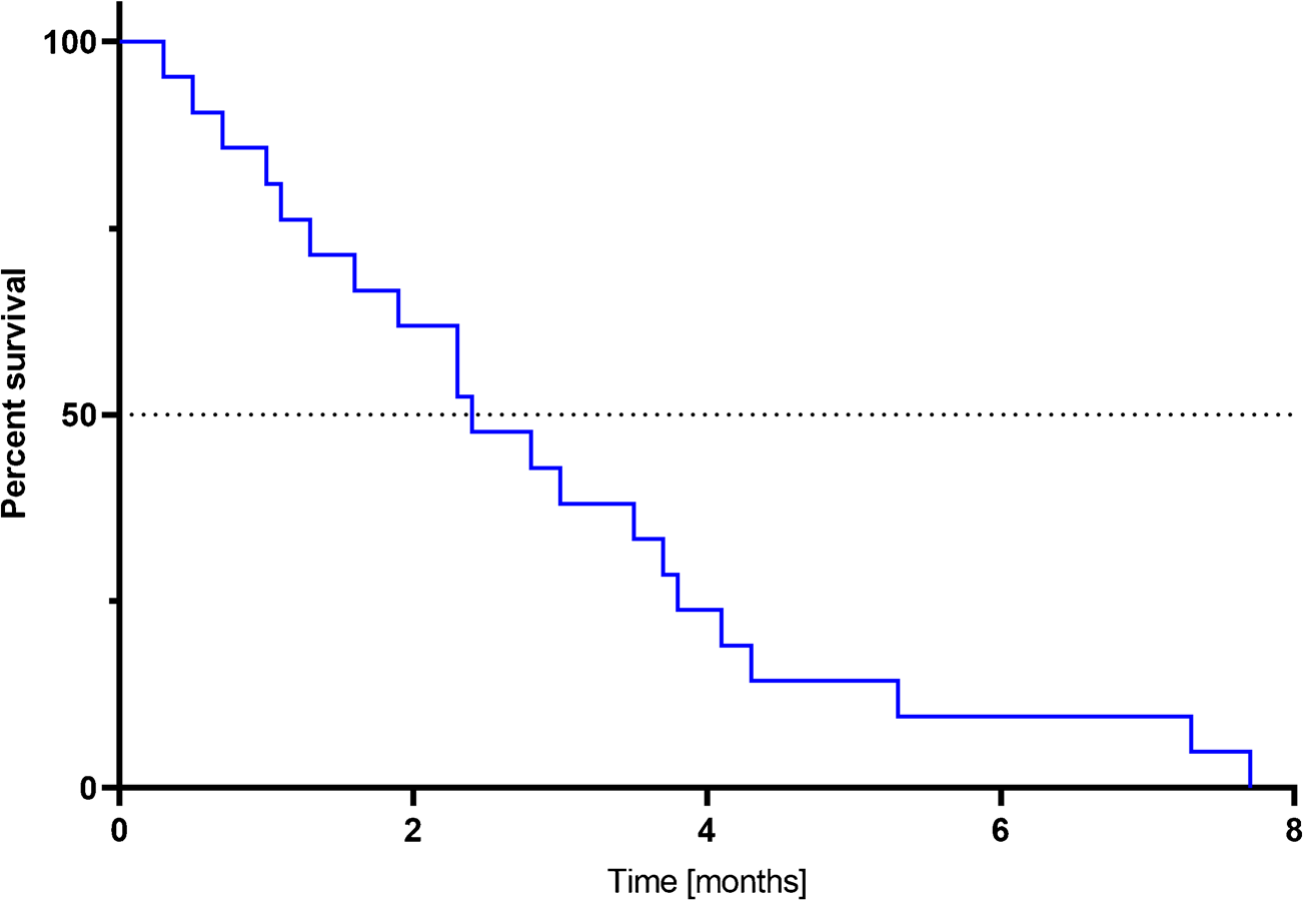
The Kaplan-Meier estimator displays the progression-free survival (PFS) in patients treated with [^225^Ac]Ac-DOTA-SP

**Fig. 4:**
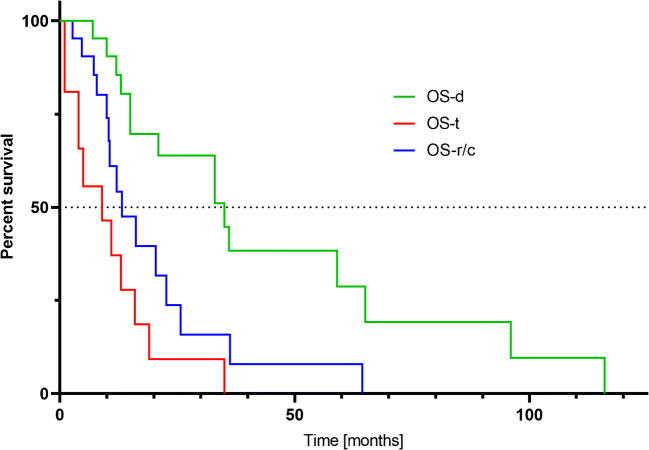
The Kaplan-Meier estimator displays survival parameters: OS-d, OS r/c and OS-t of [^225^Ac]Ac-DOTA-SP

From the start of treatment with [^225^Ac]Ac-DOTA-SP, the median PFS was 2.4 months, and the OS-t was 9.0 months. The results are summarized in the Kaplan-Meier estimator curve (Figs. 3 and 4).

During the 6 month and 12-month follow-up from the start of radioisotope treatment, 57% and 19% of patients stayed alive, respectively.

We evaluated treatment outcome in the 3 dose groups. In the group treated with 30 MBq of [^225^Ac]Ac-DOTA-SP at the time of analysis, all patients showed progression; however, 6 out of 7 were still alive with median follow-up time from the start of treatment 6.9 months, and OS parameters could be not defined.

The measured survival parameters between the 3 dose groups were not statistically significant as presented in Table 3.

**Table 3:** Survival parameters in groups treated with 10 MBq, 20 MBq and 30 MBq of [^225^Ac]Ac-DOTA-SP

	10 MBq (*n* = 4)	20 MBq (*n* = 10)	30 MBq (*n* = 7)
PFS [months]	4.5	2.6	2.3
OS-d [months]	18.0	33.0	nd
OS-t [months]	7.0	5.0	nd
OS-r/c [months]	13.0	12.5	nd

*nd*, not define

*PFS*, progression-free survival time

OS-d from the first diagnosis of the tumour to death from any cause

OS-t from the start of treatment was defined as the time from the first cycle of [^225^Ac]Ac-DOTA-SP treatment to death from any cause

OS-r/c from recurrence or conversion was defined as the time from the diagnosis of recurrence in primary tumours or conversion in secondary, to death from any cause

#### Primary vs secondary glioblastoma

We assed survival parameters also between primary and secondary glioblastoma. The secondary glioblastoma patients had statistically significant longer OS-d and OS-r/c time. Data is presented in Table 4.

**Table 4:** Survival parameters in primary and secondary glioblastoma treated with [^225^Ac]Ac-DOTA-SP

	Primary glioblastoma (*n* = 15)	Secondary glioblastoma (*n* = 6)	*p*
PFS [months]	2.4	2.9	ns
OS-d [months]	21.0	65.0	0.0125
OS-t [months]	5.0	16.0	ns
OS-r/c [months]	12.0	36.0	0.0092

*ns*, not significant

*PFS*, progression-free survival time

OS-d from the first diagnosis of the tumour to death from any cause

OS-t from the start of treatment was defined as the time from the first cycle of [^225^Ac]Ac-DOTA-SP treatment to death from any cause

OS-r/c from recurrence or conversion was defined as the time from the diagnosis of recurrence in primary tumours or conversion in secondary, to death from any cause

**Fig. 5:**
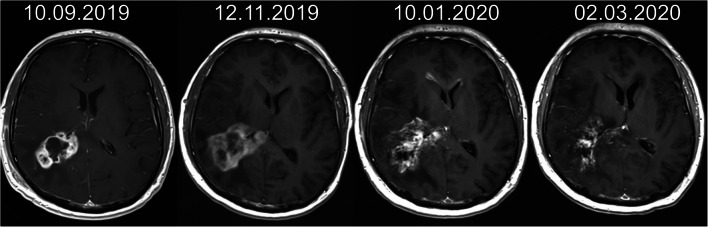
MRI images of a 42-year-old man diagnosed with recurrent glioblastoma NOS manifested 10 months after surgery and standard radiochemotherapy. The patient was treated with three cycles of 30-MBq [^225^Ac]Ac-DOTA-SP with a total activity of 90 MBq. The T1-weighed enhanced MRI image before treatment and in follow-up presented shrinkage of the tumour

The example of treatment result in MRI is shown in Fig. 5.

## Discussion

Systemic treatment of glioblastoma is ineffective and associated with numerous haematological, liver and kidney complications. Therefore, a new form of treatment should be urgently defined. Since 95% of glioblastoma manifest as unifocal lesions that recur within a 2-cm margin at the primary site [9], loco-regional treatment seems to be a promising option. Given that alpha radiation has many promising properties over beta radiation, we decided to use alpha emitters for labelling SP. At first, [^213^Bi]Bi-DOTA-SP was applied [6, 7, 10]. The results obtained were encouraging, but not satisfying. Therefore, alpha emitters with a longer half-life, which will be conducive to a better distribution of the radiopharmaceutical within the tumour, were selected for labelling of SP. ^225^Ac was used as a most useful alpha emitter since it does not depend upon generator technology like ^213^Bi.

The selection of activities of [^225^Ac]Ac-DOTA-SP was based on previous human experience with ^225^Ac-labelled radiopharmaceuticals, including [^225^Ac]Ac-DOTATOC and [^225^Ac]Ac-PSMA-617. The treatment of neuroendocrine tumours with [^225^Ac]Ac-DOTATOC was found to be safe and effective at levels of 18.5-MBq [^225^Ac]Ac-DOTATOC administered three times [11]. For treatment of metastatic, castration-resistant prostate cancer, 3 administrations of 8 MBq [^225^Ac]Ac-PSMA617 in 2-month intervals were also found safe and effective [12]. Therefore, an activity of 10-MBq [^225^Ac]Ac-DOTA-SP was selected as the initial dose and was stepwise increased to 20 MBq and 30 MBq once the lower activity levels were found to be safe without serious adverse effects.

Local adverse effects may be related to a transient increase of local pressure. Application of [^225^Ac]Ac-DOTA-SP is likely to be the cause of epileptic seizure in whom epilepsy was observed before treatment. In the study group, epilepsy was observed in 5 patients (in 4 patients after application of 30 MBq and in 1 patient after application of 20 MBq of [^225^Ac]Ac-DOTA-SP despite the antiepileptic treatment). Therefore, an adequate antiepileptic prevention is strongly recommended. An epileptic attack (epileptic status) can lead to brain oedema and seriously aggravate the patient’s clinical state. Such a case of complications was observed in 2 patients of the study group after administration of 30-MBq [^225^Ac]Ac-DOTA-SP.

If the tumour is located in the motor or speech centres, worsening of neurological symptoms should be expected after administration of the radiopharmaceutical. In the study group, transient worsening of local neurological symptoms was observed in 6 patients. These effects are usually temporary but can also be progressive in some patients.

One of the factors that could influence the results was the occurrence of satellite lesions in 8 glioblastoma patients. In all of them, the primary tumour (with an implanted catheter) was almost stable, while clinical deterioration correlated with an increase in the satellite focus. In 3 patients, a second catheter was implanted into the satellite focus, and a radiopharmaceutical was administered to both sites. The formation of satellite foci significantly worsens the prognosis, and local treatment could not prevent the manifestation of such complications. This indicates that earlier onset of therapeutic intervention, soon after completion of standard treatment should be preferred. The tumour invisibly infiltrates into deeper areas of “healthy” brain tissue and proliferates to increasing small tumour nodules until the satellites become visible on MRI. Once the intranodular pressure is getting too high, the radiopharmaceutical cannot easily penetrate anymore the nodule, because the extracellular space is closing due to pressure gradient (necrosis, decreasing oxygen levels). Early intervention targets smaller invasive cell clusters preventing satellite formation if the clusters can be saturated.

Systemic adverse reactions could be related to the pattern of vascularity in the tumour and a loss of drug to the systemic circulation. Whole-body PET examinations after intracavitary injection of [^68^Ga]Ga-DOTA-SP showed not more 1% activity in systemic circulation in proper injection. Even in cases of incorrect injection, the activity measured in the bladder was no more than 3.6%. [^225^Ac]Ac-DOTA-SP in blood was measured only in the first 2 days, due to low systemic activity. Therefore, the leakage was not monitored after that time.

The low activity in blood is most likely due to the fact that the modified, stabilized SP has a low serum half-life of only a few hours and is relatively quickly excreted via the urine. That is an advantage of the compound and limits circulation time and thus haematological toxicity. Therefore, systemic side effects should not be expected.

In one patient, transient hepatic symptoms were observed. Similar symptoms also appeared earlier immediately after chemotherapy and did not appear to be related to the radioisotope treatment. In two patients, transient thrombocytopenia grade II and III was noted which had previously already been observed during chemotherapy. In the second patient, thrombocytopenia appeared to also be related to concomitant chemotherapy. Therefore, the influence of radioisotope treatment on haematological parameters in both patients is questionable. According to our observations, the severity and frequency of side effects after loco-regional administration of the drug are considerably less significant than during systemic administration.

Our results showed that the secondary glioblastoma patients had a statistically significant longer OS-d and OS-r/c time in comparison to the patients from the primary glioblastoma group. Nevertheless, the PSF and OS-t – two of the most treatment-related parameters – were similar. This indicates that local treatment with [^225^Ac]Ac-DOTA-SP is effective in both groups of patients, regardless of the genetic background of the tumours. This is understandable given the mechanism of action of alpha radiation.

Surprisingly, the results indicate that the treatment effect of [^225^Ac]Ac-DOTA-SP was independent of the radioisotope injected activity in the range of 10 and 30 MBq. OS parameters for the group treated with 30 MBq were not defined. The OS-t in groups treated with 10 and 20 MBq ranged from 7 to 5 months, and the OS-r/c from 13 to 12.5 months. Only the OS-d was significantly shorter for patients treated with a dose of 10 MBq in comparison to patients treated with a dose of 20 MBq (18 vs 33 months). This shorter OS-d indirectly proves the aggressiveness of the tumour.

Potential cause of an effect independent of the injected activity, it is limited diffusion into the tumour, so the treatment effect is only related to absorbed dose by the peripheral area of the tumour near the cavity.

Additionally, individual differences in tumour structure should be taken into account. Heterogenity of the tissue and possible differences in diffusion of radiopharmaceutical within the tumours can be judged on MRI examination, based on the apparent diffusion coefficient (ADC, Table 1). ADC is a measure of a magnitude of diffusion of water molecules within tissues. This coefficient depends on the extent of tissue cellularity and the presence of an intact cell membrane. It is considered useful for the characterization of brain tumours [13]. ADC value in the group of patients with primary tumour ranged between 213 and 1770 mm^2^/s and in the group with the secondary glioblastoma between 825 and 1103 mm^2^/s. The diverse range of ADC values in the study group indicates possible significant differences in the diffusion of the radiopharmaceutical within tumours, which may partially explain the response to treatment.

In comparison to previously published data, survival parameters PFS and OS after treatment with [^225^Ac]Ac-DOTA-SP at the dose levels studied to date are slightly shorter compared to locoregional treatment with [^213^Bi]Bi-DOTA-SP, with a similar number and character of adverse reactions. However, with the use of [^225^Ac]Ac-DOTA-SP, the local treatment of brain tumours can be greatly simplified and does not require any additional production and distribution of the extremely costly ^213^Bi generators.

There are some limitations to this study. Even in spite of the fact that this is first study of locoregional treatment with [^225^Ac]Ac-DOTA-SP, the study population is relatively small. Therefore, more extensive research on a larger number of patients is warranted. Second, the sample size in secondary glioblastoma is smaller than that in primary glioblastoma, so the differences in the therapy-related survival parameters, PFS and OS-t between the subgroups, if any, do not reach statistical significance. Presenting survival parameters data was not dependent on injected activity, so this experience shows that local radioisotope treatment of brain tumours requires further dosimetry studies taking into account the complexity of biological processes. The next investigation should focus on how to achieve a better target saturation (injection interval, catheter position, etc).

As the disease develops, new satellite foci appear; hence, the important search for new targets and the possibility of intravenous treatment [14–16].

## Conclusions

Treatment of recurrent glioblastoma with [^225^Ac]Ac-DOTA-SP is safe and well tolerated up to 30 MBq per cycle. The escalation dose protocol showed good tolerability. Mostly mild temporary adverse effects (oedema, epileptic seizures, aphasia, hemiparesis) were observed – mainly in the group of patients treated with 30 MBq of [^225^Ac]Ac-DOTA-SP. There was no haematological, kidney or liver toxicity that could unequivocally be correlated to alpha therapy. It was related to the concomitant administration of additional medication.

More extensive studies are required to evaluate survival parameters in a larger number of patients.

## References

[1] Louis DN, Ohgaki H, Wiestler OD, Cavenee WK (2016). World Health Organization histological classification of tumours of the central nervous system. Revised.

[2] Weller M, Cloughesy T, Perry JR, Wick W (2013). Standards of care for treatment of recurrent glioblastoma-are we there yet?. Neuro-Oncology.

[3] Barbagallo GM, Jenkinson MD, Brodbelt AR (2008). 'Recurrent' glioblastoma multiforme, when should we reoperate?. Br J Neurosurg.

[4] Kneifel S, Cordier D, Good S, Ionescu MC, Ghaffari A, Hofer S (2006). Local targeting of malignant gliomas by the diffusible peptidic vector 1,4,7,10-tetraazacyclododecane-1-glutaric acid-4,7,10-triacetic acid-substance p. Clin Cancer Res.

[5] Cordier D, Forrer F, Bruchertseifer F, Morgenstern A, Apostolidis C, Good S (2010). Targeted alpha-radionuclide therapy of functionally critically located gliomas with 213Bi-DOTA-[Thi8,Met(O2)11]-substance P: a pilot trial. Eur J Nucl Med Mol Imaging.

[6] Krolicki L, Bruchertseifer F, Kunikowska J, Koziara H, Królicki B, Jakuciński M (2018). Prolonged survival in secondary glioblastoma following local injection of targeted alpha therapy with 213Bi-substance P analogue. Eur J Nucl Med Mol Imaging.

[7] Królicki L, Bruchertseifer F, Kunikowska J, Koziara H, Królicki B, Jakuciński M (2019). Safety and efficacy of targeted alpha therapy with 213Bi-DOTA-substance P in recurrent glioblastoma. Eur J Nucl Med Mol Imaging.

[8] Apostolidis C, Molinet R, Rasmussen G, Morgenstern A (2005). Production of Ac-225 from Th-229 for targeted alpha therapy. Anal Chem.

[9] Hochberg FH, Pruitt A (1980). Assumptions in the radiotherapy of glioblastoma. Neurology.

[10] Królicki L, Kunikowska J, Bruchertseifer F, Koziara H, Królicki B, Jakuciński (2020). ^25^Ac- and ^213^Bi-substance P analogues for glioma therapy. Semin Nucl Med.

[11] Kratochwil C, Bruchertseifer F, Giesel F, Apostolidis C, Haberkorn U, Morgenstern A (2015). Ac-225-DOTATOC - an empiric dose finding for alpha particle emitter based radionuclide therapy of neuroendocrine tumors. J Nucl Med.

[12] Kratochwil C, Bruchertseifer F, Rathke H, Bronzel M, Apostolidis C, Weichert W, Haberkorn U, Giesel FL, Morgenstern A (2017). Targeted alpha therapy of mCRPC with 225Actinium-PSMA-617: dosimetry estimate and empirical dose finding. J Nucl Med.

[13] Hilario A, Ramos A, Perez-Nuñez A (2012). The added value of apparent diffusion coefficient to cerebral blood volume in the preoperative grading of diffuse gliomas. AJNR Am J Neuroradiol.

[14] Kunikowska J, Królicki B, Królicki B (2018). Glioblastoma multiforme: another potential application for ^68^Ga-PSMA PET/CT as a guide for targeted therapy. Eur J Nucl Med Mol Imaging.

[15] Kunikowska J, Kuliński R, Muylle K, Koziara H, Królicki L (2020). ^68^Ga-prostate-specific membrane Antigen-11 PET/CT: a new imaging option for recurrent glioblastoma multiforme?. Clin Nucl Med.

[16] Kunikowska J, Charzyńska I, Kuliński R, Pawlak D, Maurin M, Królicki L (2020). Tumor uptake in glioblastoma multiforme after IV injection of [^177^Lu]Lu-PSMA-617. Eur J Nucl Med Mol Imaging.

